# Comparison of phenological traits, growth patterns, and seasonal dynamics of non-structural carbohydrate in Mediterranean tree crop species

**DOI:** 10.1038/s41598-019-57016-3

**Published:** 2020-01-15

**Authors:** Aude Tixier, Paula Guzmán-Delgado, Or Sperling, Adele Amico Roxas, Emilio Laca, Maciej A. Zwieniecki

**Affiliations:** 1 UMR 1347 Agroécologie, AgroSup/INRA/uB, Dijon, France; 2 Department of Plant Sciences, University of California, Davis, CA USA; 3 Department of Plant Sciences, Agriculture Research Organization (ARO), Negev, Israel

**Keywords:** Physiology, Plant sciences

## Abstract

Despite non-structural carbohydrate (NSC) importance for tree productivity and resilience, little is known about their seasonal regulations and trade-off with growth and reproduction. We characterize the seasonal dynamics of NSC in relation to the aboveground phenology and temporal growth patterns of three deciduous Mediterranean species: almond (*Prunus dulcis* (Mill.) D. A. Webb), walnut (*Juglans regia* L.) and pistachio (*Pistacia vera* L.). Seasonal dynamics of NSC were synchronous between wood tissues from trunk, branches and twigs. Almond had almost identical levels and patterns of NSC variation in twigs, branches and trunks whereas pistachio and walnut exhibited clear concentration differences among plant parts whereby twigs had the highest and most variable NSC concentration, followed by branches and then trunk. While phenology had a significant influence on NSC seasonal trends, there was no clear trade-off between NSC storage and growth suggesting that both were similarly strong sinks for NSC. A temporal trade-off observed at the seasonal scale was influenced by the phenology of the species. We propose that late senescing species experience C allocation trade-off at the end of the growing season because of C-limiting thermal conditions and priority allocation to storage in order to survive winter.

## Introduction

Rising temperatures due to global climate change are associated with significant shifts in tree phenology, while the increase in the frequency and intensity of drought events threatens their survival^1–4^. The shifts in temperature combined with drought events not only disturb non-structural carbohydrate (NSC, starch and soluble sugars) accumulation in summer but also their remobilization during winter and spring^5–8^. As remobilization of stored NSC allows plants to buffer periods of carbon (C) deficit when supply by photosynthesis is not sufficient to sustain maintenance, growth and defense, they play a key role in tree survival through periods of stress and winter dormancy and allow for resumption of growth in spring^9–15^. Hence, the disturbance of evolved seasonal patterns of NSC due to climate change may lead to an overall NSC reserve depletion, leaving trees highly vulnerable to mortality^16^. As NSC reserve depletion remains debated, another option is that strong C demand of a storage sink could reduce C supply to growth or reproduction, leading to reduction in productivity of natural populations and agroecosystems with dramatic consequences for ecosystems and food production^17–19^. It is therefore critical to understand how perennial plants integrate multiannual, seasonal and short-term NSC regulation in response to short-term stress, seasonal environmental signals and long-term global change in order to fully predict and potentially mitigate impact of climate change.

The classical view of storage formation as the accumulation of resources when supply exceeds demand is now supplanted by the understanding of storage as a competing C sink^16,20,21^. In fact, a classical model of C allocation presented as a static balance between sources and sinks is not suitable. C allocation fluctuates depending on each C-demanding function that individually responds to environmental and endogenous cues^22–24^. For example, low water potential during drought stress impedes growth prior to limiting photosynthesis, leading to increased C allocation to storage^21,25,26^. However, when intensity and/or duration of drought increase, the decline of photosynthesis can lead to re-mobilization of stored NSC to sustain metabolic needs^18,27,28^. As stress-induced allocation of C within the tree architecture is overlaid with seasonal trends bearing their own intrinsic regulations, it remains difficult to understand underlying mechanisms using only localized and momentary changes in NSC storages^12,29,30^. Hence, building a mechanistic understanding of NSC seasonal storage dynamics requires integration of short and long term interactions with other functions such as growth and reproduction.

NSC storage has seasonal fluctuations marked by the alternation between a favorable season with positive net carbon balance and a dormancy season when trees rely solely on stored NSC^10,12^. Seasonal NSC fluctuation has been reported for trees from various phylogenetic groups (gymnosperms and angiosperms), life habits (deciduous, evergreen), and biomes (Boreal, Temperate, Mediterranean and Tropical) in natural conditions^29,31–37^. Despite the variability between tree species, NSC storage remains high throughout the year (never falling below 30% of the maximum NSC concentration). Maximum and minimum NSC concentrations usually occurs around the end and at the beginning of the favorable season, respectively. Different organs (leaves, stem, roots) tend to show different patterns in NSC concentration throughout the year^17,33^. Additionally, phenology has been shown to be a key factor influencing seasonal variability of NSC concentrations^14,32,34,38^. However, we are not aware of studies with frequent-enough sampling to clearly test the impact of phenology and growth on NSC storage in the absence of abiotic and biotic stress.

The comparison of organs in species exhibiting different phenological traits and growing in the same environmental conditions allows to isolate the endogenous regulations of NSC storage. Data from drought-prone Mediterranean ecosystems are particularly scarce and were mainly collected from natural populations, showing NSC accumulation in autumn after the release from summer drought^34,39^. Seasonal dynamics of NSC in cultivated species that are not subjected to summer drought or biotic stresses should reveal patterns that are mostly determined by phenology and evolved endogenous regulation. Thus, the aim of this study was to characterize the aboveground seasonal dynamics of NSC in relation to phenology and temporal growth patterns of three deciduous Mediterranean species that are commercially grown (irrigated, fertilized and treated for biotic pests and pathogens) in the same location: almond (*Prunus dulcis* (Mill.) D. A. Webb), walnut (*Juglans regia* L.) and pistachio (*Pistacia vera* L.). We provide high temporal and spatial resolution of NSC seasonal dynamics to answer three major questions: (i) is NSC spring mobilization and accumulation at the end of the growing season synchronous in trunk, branches and twigs? (ii) can the seasonal dynamics of NSC be explained by the tree phenological status? (iii) is there a temporal trade-off between NSC accumulation and growth during the growing season?

## Material and Methods

### Seasonal NSC dynamics analysis – plant material and sample collection

**Figure 1:**
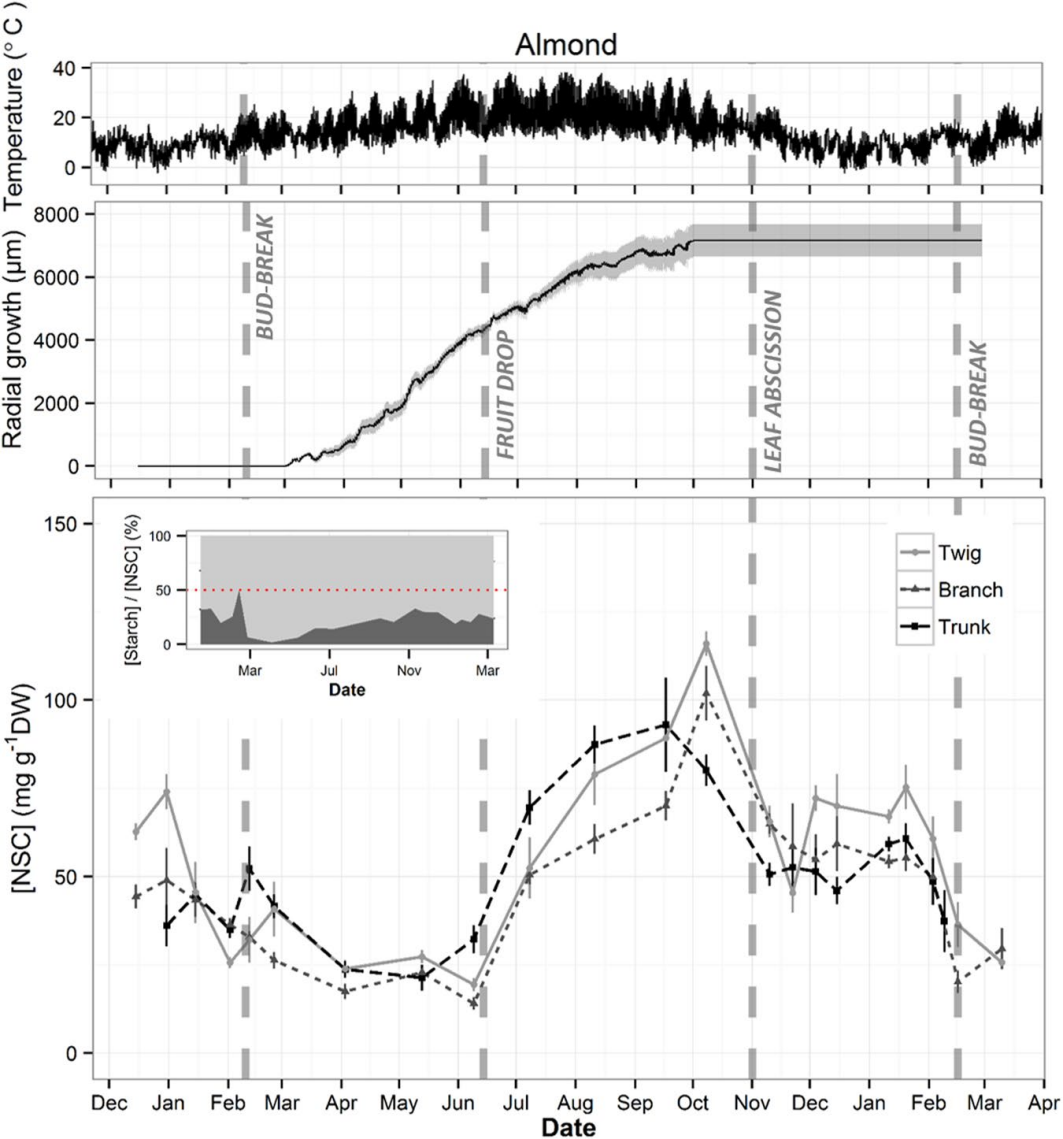
Seasonal variations of temperature, trunk radial growth and wood total non-structural carbohydrate (NSC) concentration in twig, branch and trunk in *Prunus dulcis* trees. Temperature and growth data were recorded every hour while samples for NSC analysis were collected twice a month during dormancy and once a month during the growing season. Data points represent average of five trees. Grey area in growth data and error bars in NSC data represent SE. Dashed vertical lines represent phenological events (Bud-break, fruit drop, leaf abscission). Inset in NSC plot represent the percentage of sugar (light grey) and starch (dark grey) in total NSC concentration in twigs with red dotted line showing 50%.

**Figure 2:**
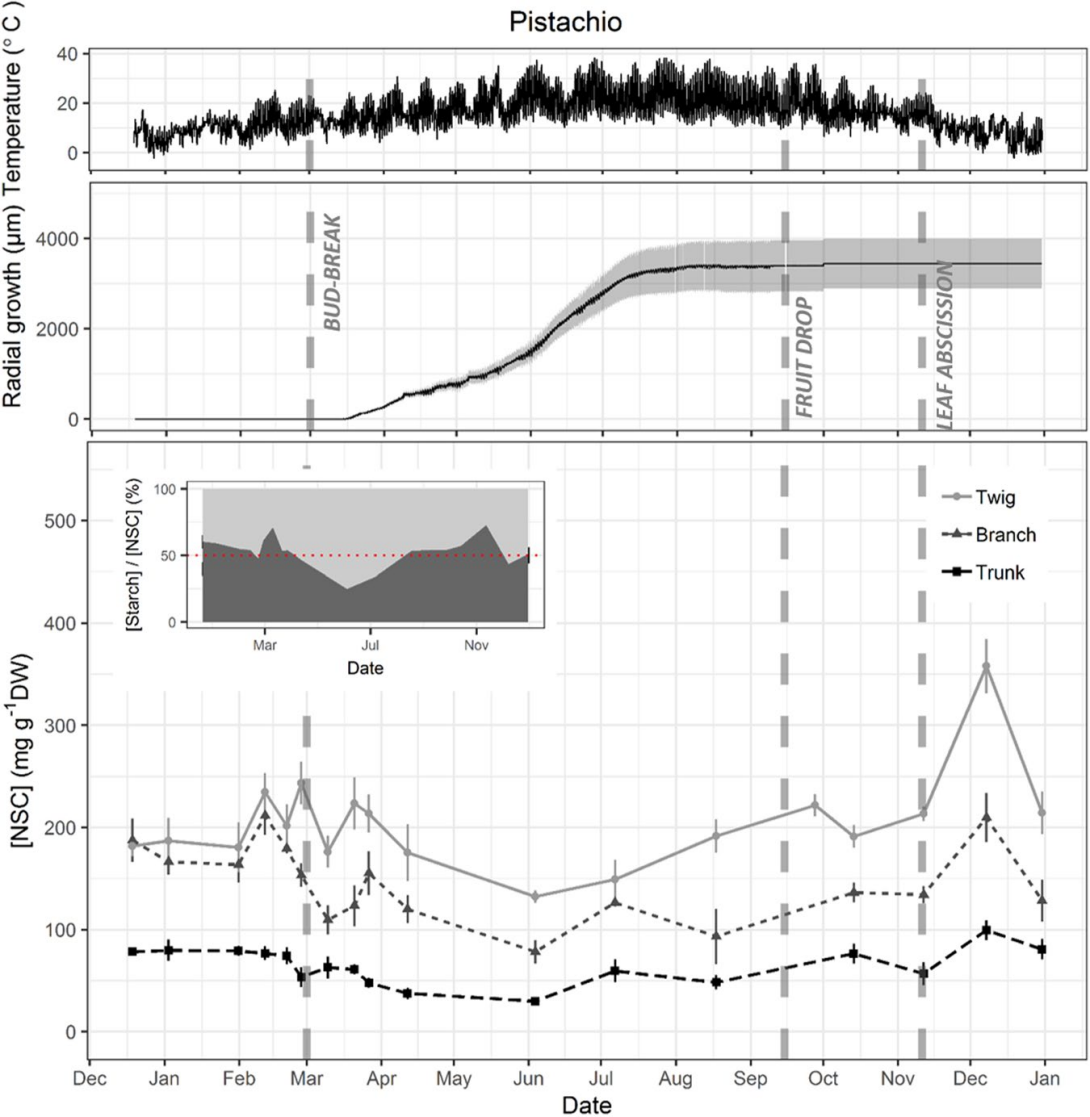
Seasonal variations of temperature, trunk radial growth and wood total non-structural carbohydrate (NSC) concentration in twig, branch and trunk in *Pistacia vera* trees. Temperature and growth data were recorded every hour while samples for NSC analysis were collected twice a month during dormancy and once a month during the growing season. Data points represent average of five trees. Grey area in growth data and error bars in NSC data represent SE. Dashed vertical lines represent phenological events (Bud-break, fruit drop, leaf abscission). Inset in NSC plot represent the percentage of sugar (light grey) and starch (dark grey) in total NSC concentration in twigs with red dotted line showing 50%.

**Figure 3:**
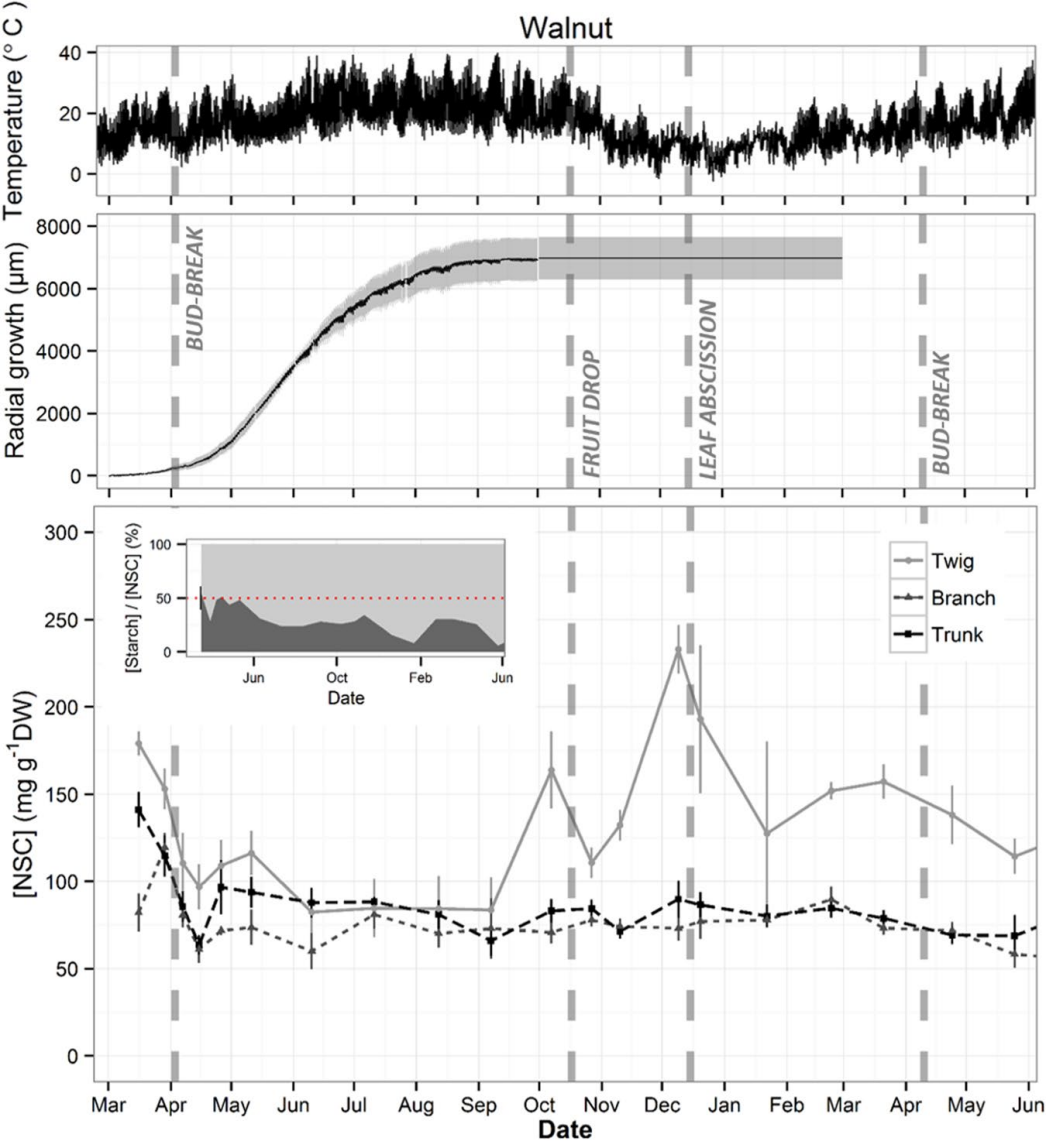
Seasonal variations of temperature, trunk radial growth and wood total non-structural carbohydrate (NSC) concentration in twig, branch and trunk in *Juglans regia* trees. Temperature and growth data were recorded every hour while samples for NSC analysis were collected twice a month during dormancy and once a month during the growing season. Data points represent average of five trees. Grey area in growth data and error bars in NSC data represent SE. Dashed vertical lines represent phenological events (Bud-break, fruit drop, leaf abscission). Inset in NSC plot represent the percentage of sugar (light grey) and starch (dark grey) in total NSC concentration in twigs with red dotted line showing 50%.

Seasonal NSC dynamics were investigated on mature trees growing in managed orchards with sprinkler irrigation system of almond (*P*. *dulcis*, root stock *P*. *persica* x *P*. *davidiana* (Nemaguard)), pistachio (*P*. *vera*, rootstock *P*. *integerrima* (J. L. Stewart ex Brandis)). and walnut (*J*. *regia*, rootstock *J*. *nigra* (Paradox)) in the vicinity of University of California, Davis. We collected twigs (3–4 mm diameter in almond and 10 mm in pistachio and walnut), branches (3 to 4-year-old, 1-cm- diameter in almond, 2-cm-long-cores from 5 cm diameter branches in pistachio and walnut) and 2-cm-long trunk cores twice a month during dormancy to characterize its NSC trends in detail and once a month during the growing season. Cores were sampled using a drill with taped drill bits to ensure reproducible depth of drilling. Five well-watered *P*. *dulcis* trees (University of California orchard: 38.5°N, −121.80°W) were sampled in 2015 and 2016 (Fig. 1). Five well-watered *P*. *vera* trees (Wolfskill Experimental Orchards: 38.5°N, −121.97°W) were sampled in 2015 (Fig. 2), and five *J*. *regia* individuals (University of California orchard: 38.5°N, −121.77°W) were sampled in 2016 and 2017 (Fig. 3). Samples were collected always at the same time of the day (10:00–11:00) to avoid diurnal variation^40^ and immediately transported to the laboratory, where bark was stripped and wood samples were dried at 75 °C for 48 hours.

### Seasonal NSC dynamics – NSC analysis

Dried wood samples were ground into a fine and homogeneous powder using a ball mill. Soluble carbohydrates (SC) were extracted by incubating 25 mg of dry powder in 1 mL of deionized water for 15 minutes at 70 °C followed by centrifugation (10 minutes at 21,000 g). The supernatant was diluted in deionized water (1:20, v/v) and SC were quantified using Anthrone as a reagent (0.1% (m/v) in 98% sulfuric acid) by reading absorbance at 620 nm. The remaining pellet was washed twice with 80% ethanol, incubated 10 min at 100 °C to allow starch gelatinization and then digested with 0.7 U of amylase and 7 U of amyloglucosidase in sodium acetate buffer (0.2 M, pH = 5.5) for 4 hours at 37 °C. Once the digestion was finished, samples were centrifuged for 10 minutes at 21,000 g. The supernatant was diluted in deionized water (1:20, v/v) and glucose obtained from starch digestion was quantified using the same method as described above.

### Phenological, temperature and growth data collection

Phenological data from the 15 trees studied for NSC concentrations were recorded for each sampling date (Figs. 1–3). Bud break was the date when full development of the first organ to develop for each species was reached, which was a flower for almond and a leaf for pistachio and walnut. Fruit abscission and leaf abscission were the dates when more than 90% of fruits or leaves were dropped, respectively. Bud break, fruit abscission and leaf abscission events were selected to represent the bounds of the 3 major phenological phases during the year. Dormancy occurred between leaf abscission and bud break and it represented the season during which trees were relying solely on storage for metabolism. The reproductive and vegetative growth phase occurred between bud break and fruit abscission, whereas the vegetative growth phase was between fruit abscission and leaf abscission.

Air temperature and radiation data were collected from the CIMIS (California Irrigation Management Information System) station at the University of California, Davis (lat.38.53°N, −121.79°W) (Figs. 1–3). Trunk radial growth data from a different set of trees were provided by Phytech Ltd. Company. Stem diameter was recorded every hour with dendrometers placed on the trunk of 5 well-watered trees for each species (Figs. 1–3). These trees were spread over the Central Valley of California and their growth can be considered representative of the trees where NSC were measured.

### Statistical analysis and modelling

The seasonal dynamics of SC, starch and NSC concentrations were described with generalized additive models (GAM) using organ and species as fixed parametric effects and tree identity as random effect. Semi-parametric terms composed of non-linear smooths functions (splines) were included for the relationships between NSC concentrations and day of year, (Doy). The p-value of the smooth term can be used to assess the significance of temporal variations. SC, starch, and NSC concentrations were log transformed to meet homogeneity of variance and normality of residuals. The optimization of smoothing parameter estimation of thin plate splines was performed with a restricted maximum likelihood (REML) method. GAM models were computed with the mgcv package (Wood, 2017) from R software^41^. In order to assess phenology effect on NSC seasonal trend, phenology phase was added to the same model as a parametric effect.

The seasonal dynamics of growth were described using logistic non-linear regression against stem radial increment (SRI, absolute increase of radius since the first measurement) using the stat package from R and the equation:

$$SRI=Max/(1+\exp )(\frac{s}{25}(Doy-{d50}))$$

where Max is the maximum stem radial increment, S is the maximum slope of the curve and d50 as the Doy where half of SRI occurred. The derivative of the function with the estimated parameters values was used to calculate growth rate. Significance for all statistical analyses was α = 0.05.

**Figure 4:**
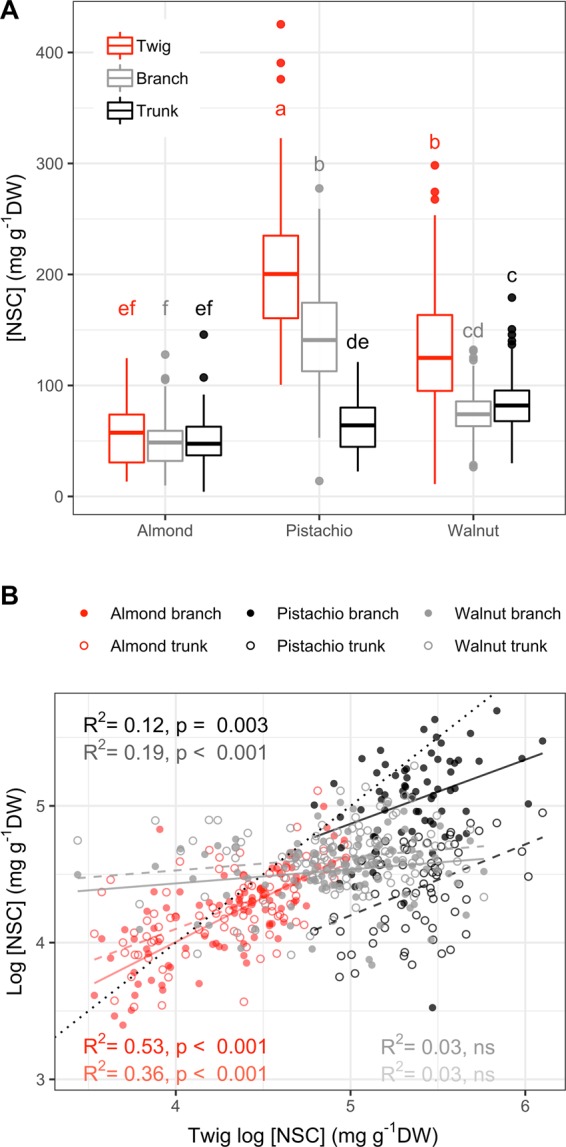
Comparison of NSC concentration between wood twig, branch trunk of almond, pistachio and walnut. Samples for NSC analysis were collected twice a month during dormancy and once a month during the growing season. Significant effect of species and sampleswas observed (**A**), ANOVA, P < 0.001). Letters represent significant groups within girdling treatment (Tukey’s HSD, P\0.05). NSC concentration of branch (solid line) and trunk (dashed line) as a function of twig NSC concentration (**B**), (log transformed). R^2^ and P-value are output of generalized linear models. Dotted line represent 1:1 line.

Data in Fig. 4A were analyzed with ANOVA considering fixed effects. Tukey’s HSD tests were performed on each model to separate means when ANOVA results were significant. Data in Fig. 4B were analyzed with generalized linear model (GLM).

## Results

### Seasonal NSC patterns

**Figure 5:**
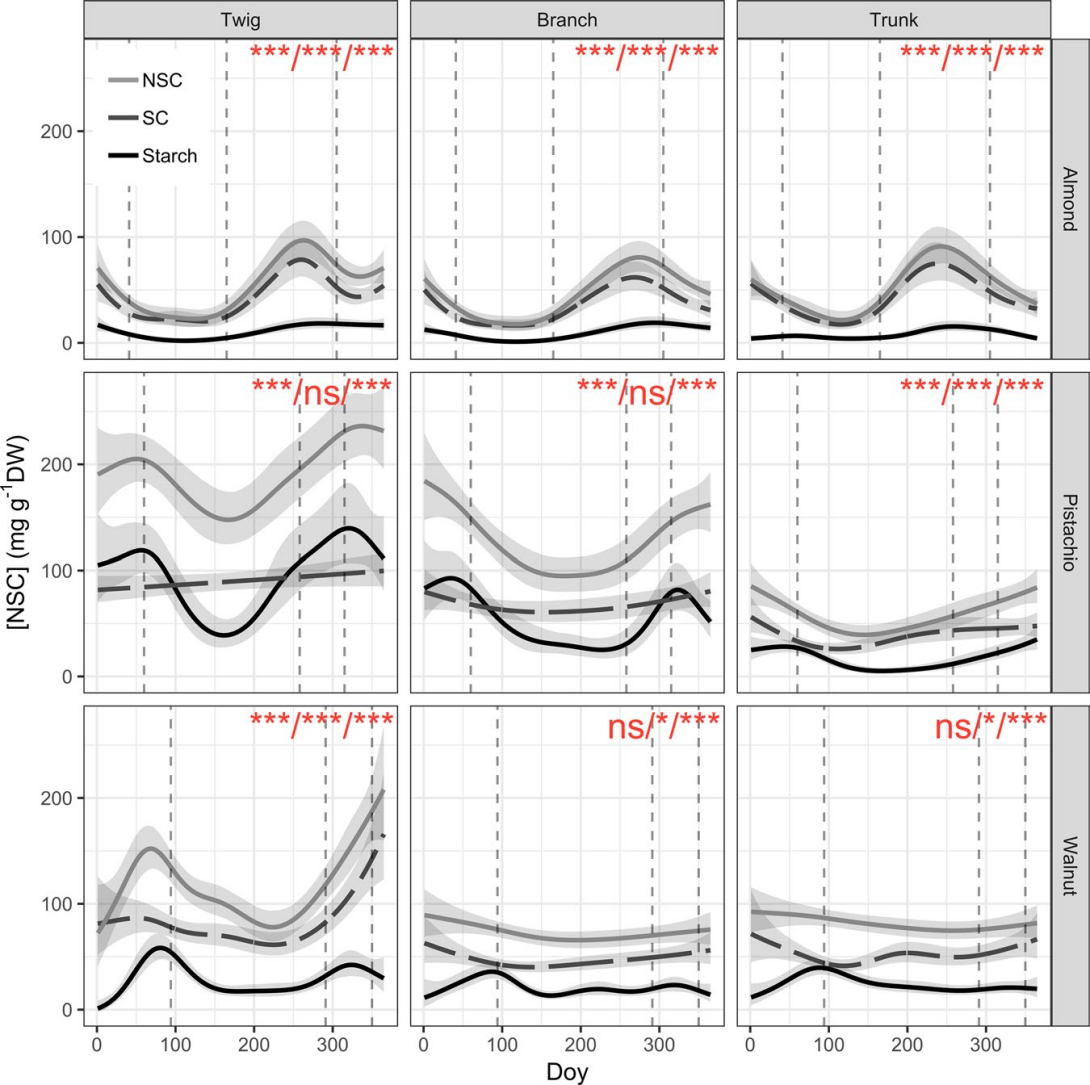
Seasonal variation of non-structural carbohydrate (NSC) concentrations as a function of day of year (Doy) for the different wood samples (columns) and species (rows) combinations, according to the fitted GAM models. Total NSC (continuous grey line), soluble carbohydrates (SC, dashed line) and starch (continuous black line) concentration are modelled from data collected on five trees and shaded areas correspond to SE. Dashed vertical lines represent phenological events (Bud-break, fruit drop, leaf abscission). In each panel, red asterisks indicate the significance of the smooth term describing Doy effect (at *P < 0.05, **P < 0.01, or ***P < 0.001 for NSC/SC/Starch, in this order; ns, not significant).

**Figure 6:**
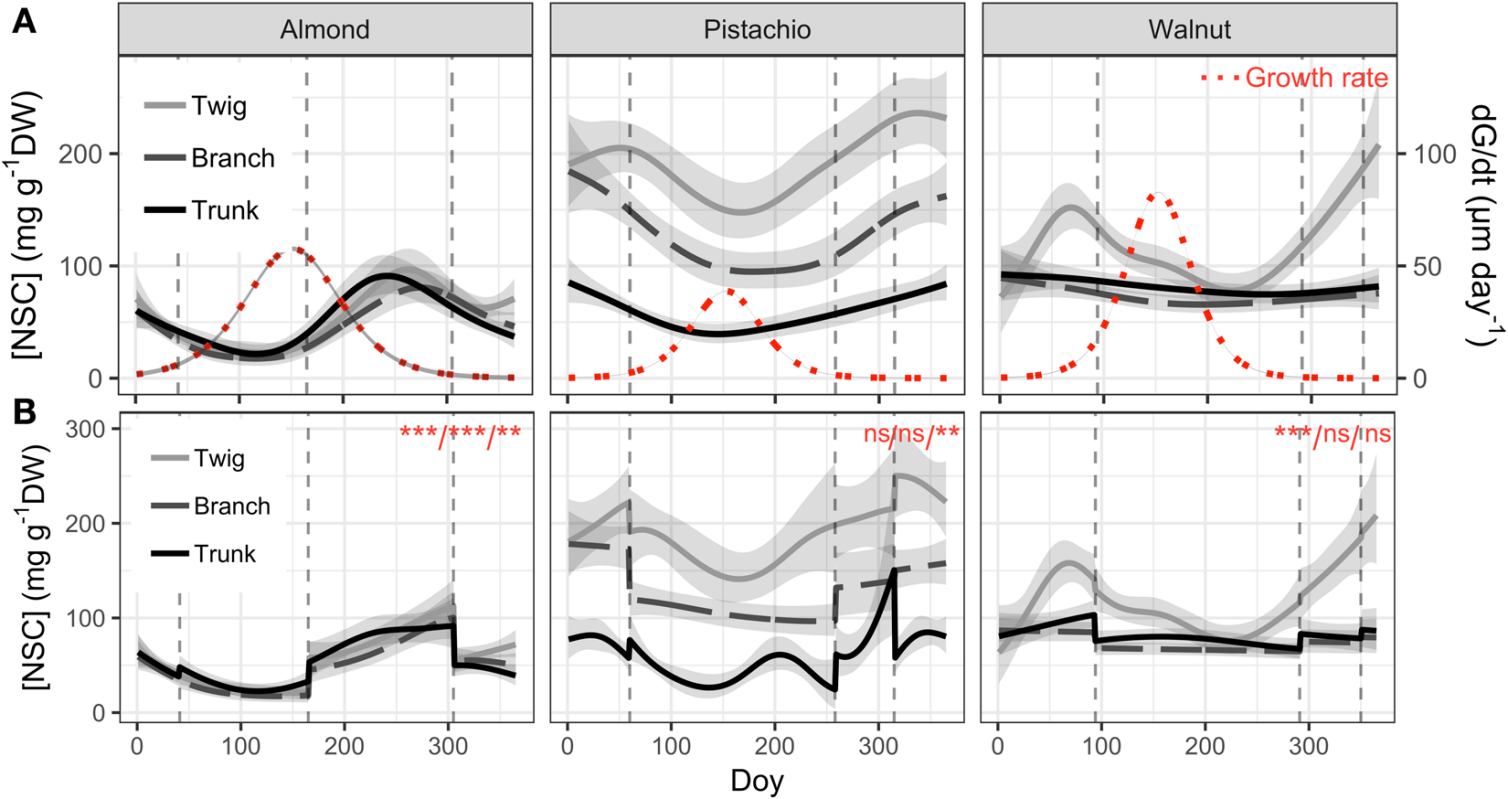
Comparison of seasonal variation of total non-structural carbohydrate (NSC) concentrations and trunk growth rate (**A**) and NSC concentrations with effect of phenology (**B**) as a function of day of for the different species (columns), according to fitted GAM models. Total NSC concentration are modelled from data collected on five trees and shaded areas correspond to SE. Red dotted lines represent growth rate modelled from data collected on five trees according to the derivative of logistic non-linear regression and shaded areas correspond to SE. Dashed vertical lines represent phenological events (Bud-break, fruit drop, leaf abscission). Phenology had a significant effect in all cases (P < 0.01). In each panel, red asterisks indicate the significance of the smooth term describing Doy effect (at *P < 0.05, **P < 0.01, or ***P < 0.001 for Twig /Branch/Trunk, in this order; ns, not significant).

Significant seasonal variation of total NSC concentration was observed in wood from all measured organs of almond and pistachio while it was only observed in twigs of walnut (Fig. 5). All three species presented the minimum total NSC concentration during the growing season whereas the maximum was in autumn (October) for almond and in winter (December) for pistachio and walnut (Figs. 1–3). Total NSC varied synchronously in twigs, branches and trunks of almond and pistachio. Total NSC concentrations were significantly different among organs in pistachio but not in almond (Fig. 4). Overall, NSC in almond organs ranged from 21.5 ± 1.5 in April to 99.3 ± 4.9 mg g^−1^ DW in October (Fig. 1). In this species, total NSC seasonal variations were the result of significant changes of SC and starch concentrations throughout the year but total NSC were mostly composed of SC (Fig. 1, inset, Fig. 5). For example, in twigs, SC concentration ranged from 60.9 ± 7.6 to 99.1 ± 0.9% of total NSC concentration in February and April, respectively (Fig. 1, inset). In pistachio, total NSC reached the highest concentrations in December with 357.9 ± 26.5, 209.7 ± 24.3 and 99.5 ± 9.6 mg g^−1^ DW for twigs, branches and trunks, respectively (Fig. 2). The lowest concentrations of total NSC were reached in June with 132.4 ± 6.1, 78.3 ± 11.6 and 29.9 ± 3.2 mg g^−1^ DW for twigs, branches and trunks, respectively. Pistachio SC concentration remained fairly constant throughout the year in twigs and branches but varied significantly in the trunk (Fig. 5). Starch showed significant concentrations fluctuations in all organs. In walnut twigs, accumulation of starch and SC during winter led to a maximum total NSC concentration of 233.1 ± 14 mg g^−1^ DW in December while minimum occurred in June (82.5 ± 13.6 mg g^−1^ DW). Total NSC concentration variation was mainly due to changes in SC (Fig. 3). In branches and trunks, significant seasonal variation of SC and starch did not lead to significant changes in total NSC concentration which fluctuated throughout the year around 74.5 ± 2 and 84.4 ± 2.4 mg g^−1^ DW in branches and trunk, respectively (Fig. 6A). Pistachio and walnut displayed a spatial gradient of NSC concentration, increasing from trunk to twigs (Figs. 4 and 6A). While the trunk total NSC concentrations were similar in the 3 studied species (maximum reaching 93.0 ± 13.3, 99.5 ± 9.6 and 89.8 ± 10.68 mg g^−1^ DW in almond, pistachio and walnut, respectively), they exhibited different ranges of twig wood total NSC concentrations throughout the year, with the highest levels observed in pistachio and the lowest in almond (Figs. 1–3, 4).

Species differed dramatically in the relationships between concentration of NSC in different organs over time. Whereas NSC concentrations in twigs, branches and trunk were positively correlated in almond and pistachio no correlation were observed in walnut. As starch concentration increased, the difference between twig and trunk increased. This may reflect different strategies of NSC storage and translocation among species.

### Effect of phenology on NSC seasonal trends

While the duration of the growing season was similar in almond, pistachio and walnut lasting 264, 255 and 256 days respectively, phenological events occurred at different time for each species (Figs. 1–3). Bud-break was completed on February 10^th^, March 1^st^ and April 3^rd^, the end of reproductive and vegetative growth phase on June 14^th^, September 15^th^ and October 17^th^ while leaf abscission on November 1^st^, November 11^th^ and December 15^th^ for almond, pistachio and walnut, respectively. Adding phenology as a parametric factor for GAM model validated its significant effect on NSC seasonal trends in all three species although it did not explained exclusively the temporal variability of seasonal NSC trends (Fig. 6B).

Bud break was consistently followed by a decrease of starch and total NSC concentration except for trunk of pistachio while the end of the reproductive and vegetative growth phase was tailed by an increase in total NSC concentration for twigs, branches and trunks of the three species (Fig. 6B). At the end of reproductive growth, total NSC are lowest and fruits cease to be a major C demand for carbohydrates. Almonds and pistachios exhibited increases in both starch and SC, whereas walnuts exhibited a sharp increase in total NSC of twigs. At the end of the growing season, which was marked by leaf abscission, a decrease in total NSC concentration in the three organs was observed in almond, while in walnut it marked an increase in total NSC concentration. In pistachio, leaf abscission was the starting point of a decrease in trunk total NSC concentration while concentration increased in twigs and branches (Fig. 6B).

### Radial growth and NSC seasonal trends

**Figure 7:**
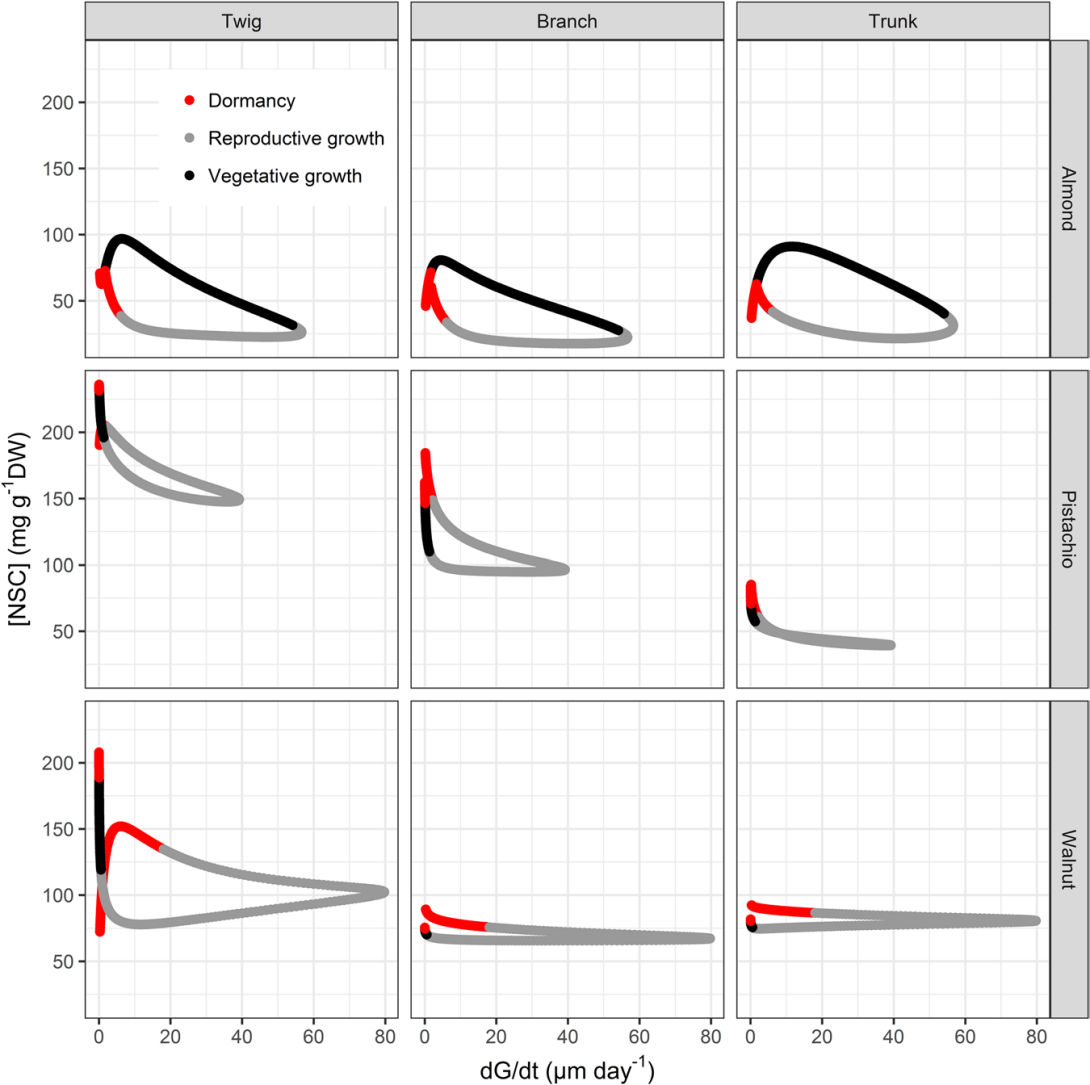
Seasonal variation of total non-structural carbohydrate (NSC) concentrations and for the different wood samples (columns) and species (rows) combinations as a function of trunk radial growth rate. NSC data are fitted GAM models from data collected on five trees and logistic regression of trunk growth from data collected on five trees (see methods). Phenological phases are represented in red, grey and black for dormancy, reproductive growth and vegetative growth, respectively.

Trunks grew only during the growing season and reached maximum growth rate in June for the 3 species (56.6 ± 3.5, 39.2 ± 0.2 and 79.7 ± 0.3 µm day^−1^, for almond, pistachio and walnut, respectively (Fig. 6)). While almond exhibited a positive radial growth rate during its entire growing season (Figs. 1, 4), pistachio and walnut stopped growth well before fruit drop and at least 4 months before leaf abscission (Figs. 2, 3, and 6). The higher growth rate in walnut and longer growing period of almond led to comparable total trunk radial increment for both species with 6933.2 ± 1.4 µm and 7112.6 ± 2.1 µm SRI for walnut and almond, respectively. Pistachio had lower growth rate and shorter growing period, reaching a total of 3467 ± 1.2 µm SRI. While the lower radial increment in pistachio trunk was associated with highest levels of NSC in the canopy, walnut showed higher levels of NSC than almond for similar radial increment but higher growth rate. The magnitude of seasonal fluctuations of NSC concentrations and trunk growth rates did not indicate a general trade-off between the two functions. For instance, while higher growth rate was associated with lower NSC seasonal fluctuation in walnut, the slower growth in pistachio trunk showed lower NSC seasonal fluctuation than almond (Fig. 7).

We related NSC and growth rate in order to assess potential temporal trade-off at the seasonal scale between the two functions (Fig. 7). Minimum NSC levels coincided with maximum growth rate while maximum NSC levels occurred when growth was low or null in all samples of the three species studied except for walnut. In that species, no significant NSC seasonal variation was observed in branches and trunk. In walnut twigs, both minimum and maximum NSC levels occurred at low growth rate although low NSC concentrations were also observed at maximum growth rate. When growth commenced in spring, NSC declined while growth rate increased in the three species (Fig. 7). During this phase of growth rate increase, the NSC level stabilized in twigs and branches of almond. After maximum growth occurred, decrease in growth rate was associated with accumulation of NSC or stabilization of values in the all three species. Almond, trunks and twigs of pistachio showed accumulation of NSC as growth rate decreased while stabilization of values was observed for branches of pistachio, branches and trunk of walnut. The later cases of NSC concentration stabilization was followed by accumulation of NSC only once growth rate reached a null value. These phases of NSC accumulation coincided with the phenological shift from reproductive and vegetative growth to vegetative growth only (Fig. 7).

## Discussion

### NSC seasonal trend synchronism and spatial gradients in wood tissues

The three species exhibited distinct patterns of NSC variation, with high synchronicity among studied woody organs but different concentrations (Fig. 5). Almond had almost identical levels and patterns of seasonal NSC variation in twigs, branches and trunks. Pistachios exhibited identical patterns of seasonal NSC variation but clear differences of concentration among plant parts whereby twigs had the highest level and most variation in total NSC concentration, followed by branches and then the trunk. Walnuts exhibited NSC variation mostly in twigs, with total NSC levels in branches and trunk exhibiting only mild seasonal variation. In trunk, the three species exhibited similar maximum NSC levels at the end of the growing season (Fig. 5). The frequent sampling presented in this study allowed to clearly identify seasonal trends in the three studied species with reliable maxima and minima as advocated by recent work^37^.

The total NSC concentrations in twigs varied dramatically throughout the year in the three species. Twigs’ total NSC accumulation prior to dormancy and its drop during bud break (Fig. 5) might reflect the temporal importance of these pools for developing buds, thus potentially linking twig total NSC content to fruit bearing capacity^42^. The higher NSC levels in twigs might be accumulated on low fruit bearing years to sustain higher number of floral buds for the next high fruit bearing year^32,42^. The observations of these spatial gradients of total NSC concentration in pistachio and almond trees during low fruit bearing years and their absence during high fruit bearing years support this hypothesis^40,43^. In the context of increasing winter temperatures and higher frequency of frost-thaw events, higher NSC demand during dormancy may overall lead to a general reduction of these crucial reserves for spring growth which would weaken reproduction capacity and growth^44^.

The high level of NSC dynamics in twigs and its synchronism with NSC level in branches and trunk suggests that the relatively non-invasive collection of twigs in the field might be a good alternative to assess aboveground NSC seasonal variation without having to extract cores from trunks. However, as the quantitative relationships between concentrations in twigs and other organs appears to be species-specific a total analysis of NSC using twigs only should be preceded with all tissue sampling. Using twig only approach would offer several benefits. (1) Provide insight to most dynamic storage compartment where high concentration fluctuations of NSC aren’t hindered by long-term storage^45^ (Fig. 4) as evidenced by the stable NSC pools in trunk throughout the year in walnut (Fig. 5) which are also reported in many studies and can be attributed to long-term storage that ensure survival during stress^10,29,32,34,45–49^. (3) Avoid injuries as collecting cores can lead to stem splitting and fungi infections, as it is known to happen in walnut. (4) Provide an option for large scale ecological or horticultural studies on NSC seasonal dynamics that can follow individual trees over prolonged periods of time and frequent sampling.

### Phenology and non-structural carbohydrates

Phenology is usually characterized by empirical discrete events that translate continuous seasonal dynamics into discrete stages. Elucidating the link between phenology and physiology is challenging, because physiological processes are practically continuous and may be linked to visible phenological events by complex combinations of rates and thresholds. For example, in accordance with the literature, bud break was followed by a decrease in NSC concentration and fruit drop was tailed by an increase in total NSC concentration (Fig. 4)^14,32,34,38^. Similarly, fruit production was always associated with low NSC levels in the three species which suggests that fruit production takes precedence over storage (Figs. 1–3)^50,51^. Prior to leaf abscission (Fig. 6), there was always increase in total NSC concentration in twigs that was either maintained (pistachio) or dropped to a lower level (almond, walnut) possibly due to mobilization for metabolism, translocation to roots^35,52^. This accumulation of NSC at leaf abscission is most likely associated with the storage of NSC to ensure winter survival^8,10,32,35,50^.

It is worth noting that the phenological events do not mark shifts in accumulation and mobilization rates (derivative of the NSC trend). This might be due (i) to the fact that the visual assessment of phenological shifts is always delayed compared to the shifts *in planta* when NSC mobilization or accumulation rate change and (ii) that effect of phenological events are buffered by the compartment size, meaning that larger organ size and higher relative contribution of storage cells in wood would attenuate any sudden response and explain discrepancies between species and organs^44,53^. Indeed, higher storage capacity infers higher inertia and momentum^15^. While the use of twigs limits these issues for studying NSC seasonal trend in response to environmental and endogenous cues, the use of dynamic modelling taking NSC fluxes and compartment sizes in consideration will be required to understand whole tree NSC seasonal dynamics.

### NSC storage and growth

Maximum growth rate occurred at the same time of the year for all three species, despite growth starting and stopping at different time of the year (Fig. 6). Maximum growth rate coincided with high temperatures (~30 °C) and long days which might represent optimal conditions for growth while the three species exhibit different temperature thresholds for cambial initiation^24,54,55^. While trunk growth start generally coincided with bud-break for the studied species, trunk growth arrest occurred much earlier than leaf abscission in pistachio and walnut (Figs. 1–3). This growth arrest concomitant with the accumulation of NSC levels at the end of growing season (Figs. 6 and 7), can neither be attributed to water stress or to low temperatures^21,25,26^, as these trees were irrigated and experienced permissive temperatures (~20 °C). Our results rather suggest that allocation to build reserves before dormancy is prioritized over growth, as an evolved seasonal pattern to ensure winter survival. Potential tradeoffs with C assimilation, underground growth and reserves formation should be addressed in the future to assess C allocation at the whole tree level.

In addition, we did not observe any trade-off between total NSC levels and magnitude of seasonal allocation toward growth. Species bearing higher levels of NSC or accumulating more NSC at the seasonal scale didn’t necessarily exhibit greater radial increment or growth rate (Fig. 7). These results are in line with reports showing that negative correlation between growth and storage are not observed in every species^21^. Yet, maxima NSC were observed at minimum growth rate and *vice versa* at the seasonal scale^24^. C allocation to storage in pistachio and walnut coincided with growth arrest which suggests that C storage is given priority over growth at the end of the growing season when environmental conditions are more limiting for C assimilation. Indeed, these species experienced lower temperatures during the vegetative growth than almond (Figs. 1–3). The resulting phenology would hence mark an angular L-shape growth/storage relationship where NSC accumulation only occurs as radial growth stops (Fig. 7). On the other hand, the earlier bloomer almond species experienced optimum C assimilation conditions after reproductive growth, allowing for concomitant NSC allocation towards reserves and growth which resulted in the elliptic growth/storage relationships (Fig. 7). The counterpart of this early phenology strategy is that bud break, growth initiation and in general, reproductive growth, befall in C limiting conditions (lower temperatures), fostering important storage mobilization to buffer C demands. These observations suggest that the context of phenology, time of year and associated environmental conditions are crucial when studying trade-off in response to C limiting stress.

### Conclusions and perspectives

This study provides high temporal seasonal dynamics of NSC for almond, pistachio and walnut. It highlights synchronism of NSC dynamics in the canopy with no preferential site of accumulation (twigs, limbs, trunk). This finding provides exciting perspectives for the use of twigs at larger scales to allow for high frequency, low impact sample collection. The integration of environmental and interspecific variability will offer means to understand the relative importance of internal and external factors on NSC seasonal dynamics and we argue for the comparison of natural and horticultural systems that would allow to determine climate shift impact on NSC storage and phenology independent of water and nutrient stress.

## References

[1] Ford Kevin R., Harrington Constance A., Bansal Sheel, Gould Peter J., St. Clair J. Bradley (2016). Will changes in phenology track climate change? A study of growth initiation timing in coast Douglas-fir. Global Change Biology.

[2] Field, C. B. *et al*. *Climate Change 2014: Impacts*, *Adaptation*, *and Vulnerability*. *Summaries*, *Frequently Asked Questions*, *and Cross-Chapter Boxes*. *A contribution of Wroking Group II to the Fifth Assessment Report of the Intergovernmental Panel on Climate Change*. (2014).

[3] Luedeling E, Guo L, Dai J, Leslie C, Blanke MM (2013). Differential responses of trees to temperature variation during the chilling and forcing phases. Agric. For. Meteorol..

[4] Choat B (2012). Global convergence in the vulnerability of forests to drought. Nature.

[5] Hartmann Henrik, Ziegler Waldemar, Kolle Olaf, Trumbore Susan (2013). Thirst beats hunger - declining hydration during drought prevents carbon starvation in Norway spruce saplings. New Phytologist.

[6] Tixier A (2017). Role of Bark Color on Stem Temperature and Carbohydrate Management during Dormancy Break in Persian Walnut. J. Am. Soc. Hortic. Sci..

[7] Pagter M, Andersen UB, Andersen L (2015). Winter warming delays dormancy release, advances budburst, alters carbohydrate metabolism and reduces yield in a temperate shrub. AoB Plants.

[8] Charrier G, Ngao J, Saudreau M, Améglio T (2015). Effects of environmental factors and management practices on microclimate, winter physiology, and frost resistance in trees. Front. Plant Sci..

[9] Bonhomme M (2009). (Carbohydrate uptake from xylem vessels and its distribution among stem tissues and buds in walnut (*Juglans regia* L.). Tree physiology.

[10] Sperling O (2019). Predicting bloom dates by temperature mediated kinetics of carbohydrate metabolism in deciduous trees. Agricultural and Forest Meteorology.

[11] Hoch G, Richter A, Korner C (2003). Non-structural carbon compounds in temperate forest trees. Plant Cell Environ..

[12] Gough CM, Flower CE, Vogel CS, Dragoni D, Curtis PS (2009). Whole-ecosystem labile carbon production in a north temperate deciduous forest. Agric. For. Meteorol..

[13] Hoch G, Körner C (2012). Global patterns of mobile carbon stores in trees at the high-elevation tree line. Glob. Ecol. Biogeogr..

[14] Sperling, O., Earles, J. M., Secchi, F., Godfrey, J. & Zwieniecki, M. A. Frost induces respiration and accelerates carbon depletion in tree*s*. *Plos One* **10** (2015). 10.1371/journal.pone.0144124 PMC4668004 26629819

[15] Tixier A (2017). Spring bud growth depends on sugar delivery by xylem and water recirculation by phloem Münch flow in *Juglans regia*. Planta.

[16] Hartmann H, Trumbore S (2016). Understanding the roles of nonstructural carbohydrates in forest trees - from what we can measure to what we want to know. New Phytol..

[17] McDowell NG (2011). Mechanisms linking drought, hydraulics, carbon metabolism, and vegetation mortality. Plant Physiol..

[18] Sala A (2009). Lack of direct evidence for the carbon-starvation hypothesis to explain drought-induced mortality in trees. Proc. Natl. Acad. Sci..

[19] Sala A, Hoch G (2009). Height-related growth declines in ponderosa pine are not due to carbon limitation. Plant, Cell Environ..

[20] Woodruff DR (2015). Linking nonstructural carbohydrate dynamics to gas exchange and leaf hydraulic behavior in Pinus edulis and Juniperus monosperma. New Phytol..

[21] Sala A, Woodruff DR, Meinzer FC (2012). Carbon dynamics in trees: Feast or famine?. Tree Physiol..

[22] Palacio S, Hoch G, Sala A, Körner C, Millard P (2014). Does carbon storage limit tree growth?. New Phytol..

[23] Sala A (2017). Physiological mechanisms of drought-induced tree mortality are far from being resolved. New Phytol..

[24] Stitt M, Zeeman SC (2012). Starch turnover: pathways, regulation and role in growth. Curr. Opin. Plant Biol..

[25] Körner C (2015). Paradigm shift in plant growth control. Curr. Opin. Plant Biol..

[26] Woodruff DR, Meinzer FC (2011). Water stress, shoot growth and storage of non-structural carbohydrates along a tree height gradient in a tall conifer. Plant, Cell Environ..

[27] Ryan MG (2010). Editorial: Temperature and tree growth. Tree Physiol..

[28] Bréda N, Huc R, Granier A, Dreyer E (2006). Temperate forest trees and stands under severe drought: a review of ecophysiological responses, adaptation processes and long-term consequences. Ann. For. Sci..

[29] Earles JM, Stevens JT, Zwieniecki MA (2018). Extreme mid-winter drought weakens tree hydraulic – carbohydrate systems and slows growth. New Phytol..

[30] Richardson AD (2013). Seasonal dynamics and age of stemwood nonstructural carbohydrates in temperate forest trees. New Phytol..

[31] Plavcova L, Hoch G, Morris H, Ghiasi S, Jansen S (2016). The amount of parenchyma and living fibers affects storage of nonstructural carbohydrates in young stems and roots of temperate trees. Am. J. Bot..

[32] Regier N, Streb S, Zeeman SC, Frey B (2010). Seasonal changes in starch and sugar content of poplar (Populus deltoides x nigra cv. Dorskamp) and the impact of stem girdling on carbohydrate allocation to roots. Tree Physiol..

[33] Barbaroux C, Bréda N (2002). Contrasting distribution and seasonal dynamics of carbohydrate reserves in stem wood of adult ring-porous sessile oak and diffuse-porous beech trees. Tree Physiol..

[34] Martinèz-Vilalta J (2016). Dynamics of non- structural carbohydrates in terrestrial plants: a global synthesis. Ecol. Monogr..

[35] Palacio S (2018). Are storage and tree growth related? Seasonal nutrient and carbohydrate dynamics in evergreen and deciduous Mediterranean oaks. Trees.

[36] Klein T, Vitasse Y, Hoch G (2016). Coordination between growth phenology and storage in the three coexisting deciduous tree species in a temperate forest. Tree physiol..

[37] Furze ME (2019). Whole-tree nonstructural carbohydrate storage and seasonal dynamics in five temperate species. New Phytol..

[38] Palacio S, Maestro M, Montserrat-Martí G (2007). Seasonal dynamics of non-structural carbohydrates in two species of mediterranean sub-shrubs with different leaf phenology. Environ. Exp. Bot..

[39] Rosas T, Galiano L, Ogaya R, Peñuelas JJ, Martínez-Vilalta J (2013). Dynamics of non-structural carbohydrates in three Mediterranean woody species following long-term experimental drought. Front. Plant Sci..

[40] Tixier A, Orozco J, Amico Roxas A, Earles JM, Zwieniecki MA (2018). Diurnal variation in non-structural carbohydrate storage in trees: remobilization and vertical mixing. Plant Physiol..

[41] R Core Team. R: A language and environment for statistical computing. *R Foundation for Statistical Computing*, *Vienna*, *Austria*. *ISBN 3-900051-07-0*, *URL* (2013). Available at, http://www.r-project.org/.le.

[42] Fernandez E (2018). Fruit load in almond spurs define starch and total soluble carbohydrate concentration and therefore their survival and bloom probabilities in the next season. Sci. Hortic. (Amsterdam)..

[43] Spann, T. M., Beede, R. H. & Dejong, T. M. Seasonal carbohydrate storage and mobilization in bearing and non-bearing pistachio (Pistacia vera) trees. 207–213 (2008). 10.1093/treephys/28.2.207 18055431

[44] Tixier A, Gambetta GA, Godfrey J, Orozco J, Zwieniecki MA (2019). Non-structural Carbohydrates in Dormant Woody Perennials; The Tale of Winter Survival and Spring Arrival. Frontiers (Boulder)..

[45] Keel SG, Siegwolf RTW, Jäggi M, Körner C (2007). Rapid mixing between old and new C pools in the canopy of mature forest trees. Plant, Cell Environ..

[46] Richardson AD (2015). Distribution and mixing of old and new nonstructural carbon in two temperate trees. New Phytol..

[47] Lacointe A (1993). Mobilization of carbon reserves in young walnut trees. Acta Bot. Gall..

[48] Höll, W. Storage and mobilization of carbohydrates and lipids. in *Trees - Contributions to Modern Tree Physiology* (eds. Rennenberg, H., Eschrich, W. & Ziegler, H.) 197–211 (Backhuys Publishers, 1997).

[49] Scartazza A, Moscatello S, Matteucci G, Battistelli A, Brugnoli E (2013). Seasonal and inter-annual dynamics of growth, non-structural carbohydrates and C stable isotopes in a Mediterranean beech forest. Tree Physiol..

[50] York N, Garden B (2011). Sources and Sinks in Woody Plants Carbohydrate. New York.

[51] Da Silva D, Qin L, Debuse C, Dejong TM (2014). Measuring and modelling seasonal patterns of carbohydrate storage and mobilization in the trunks and root crowns of peach trees. Ann. Bot..

[52] Sperling O, Silva LCR, Tixier A, Théroux-Rancourt G, Zwieniecki MA (2017). Temperature gradients assist carbohydrate allocation within trees. Sci. Rep..

[53] Morris H (2015). A global analysis of parenchyma tissue fractions in secondary xylem of seed plants. New Phytol..

[54] Begum S, Nakaba S, Yamagishi Y, Oribe Y, Funada R (2013). Regulation of cambial activity in relation to environmental conditions: Understanding the role of temperature in wood formation of trees. Physiol. Plant..

[55] Begum S (2018). Climate change and the regulation of wood formation in trees by temperature. Trees - Struct. Funct..

